# Atrial Tachycardias After “Multiple” Previous Ablations for Tachyarrhythmias: Treatment by Anti-arrhythmic Drugs or Additional Ablation?

**DOI:** 10.19102/icrm.2024.15037

**Published:** 2024-03-15

**Authors:** Bharat K. Kantharia, Zaw Win Tun, Arti N. Shah

**Affiliations:** 1 Cardiovascular and Heart Rhythm Consultants, New York, NY, USA; 2 Icahn School of Medicine at Mount Sinai, New York, NY, USA; 3 Mount Sinai Hospital—Morningside, New York, NY, USA; 4 NYC Health and Hospitals, Elmhurst, Queens, NY, USA

**Keywords:** Atrial fibrillation, atrial tachyarrhythmias, catheter ablation, electroanatomic CARTO^®^ mapping, high-density mapping

## Abstract

Pulmonary vein (PV) isolation (PVI) ablation as the first-line therapy for atrial fibrillation (AF) and repeat PVIs for patients who had symptomatic improvement with the index PVI but who develop AF recurrence are directed by practice guidelines. How many catheter ablation (CA) procedures constitute the definition of “multiple” ablations is not known. Whether atrial tachyarrhythmias (AF, atrial tachycardia [AT], atrial flutter [AFL]) that occur post-ablation are due entirely to the proarrhythmic effects of CA or a continuum of the arrhythmia spectrum from the underlying atriopathy is debatable. Herein, we describe a case of a 65-year-old man with a CHA_2_DS_2_-VASc score of 5 points who suffered from atrial tachyarrhythmias for which seven CA procedures were performed. Because of symptomatic and drug-refractory AT/AFL that failed cardioversions, he requested another ablation procedure. During the eighth procedure, high-density three-dimensional electroanatomic mapping, including Coherent and Ripple mapping (CARTO^®^ 3; Biosense Webster, Diamond Bar, CA, USA), of AT/AFL was performed. Small discrete areas of relatively viable tissue within an extensively scarred left atrium and a macro–re-entrant circuit with early-meets-late activation between the left atrial anterior wall and the right superior PV were found. Radiofrequency ablation performed at this site resulted in the termination of the tachycardia, and bidirectional conduction block across the line was achieved. On clinical follow-ups and rhythm monitoring by an implantable loop recorder, the patient remained in sinus rhythm with significant clinical improvement. Our case suggests that, in patients with prior multiple CAs, additional clinically indicated ablation should be performed using high-density mapping to accurately identify arrhythmia mechanisms, elucidate the disease substrate, and restore the sinus rhythm successfully.

## Introduction

Atrial fibrillation (AF), with its sustained global epidemic, is the most common arrhythmia encountered in daily practice. Non-pharmacological treatments, ie, anti-arrhythmic drugs (AADs) and catheter ablation (CA), are established rhythm-control modalities to treat AF.^1–4^ Various professional societies recommend pulmonary vein (PV) isolation (PVI) ablation, which forms the mainstay of AF ablation as the first-line therapy for AF, and even repeat PVIs for those patients who develop AF recurrence but had symptomatic improvement with initial PVI.^1–4^ In patients with persistent and long-standing persistent AF, more extensive ablation beyond PVI has been advocated for.^1–4^ Furthermore, to treat recurrent atrial tachyarrhythmias (AF, atrial tachycardia [AT], atrial flutter [AFL]); linear lesions in the atria; isolation of the left atrial appendage (LAA) or of the superior vena cava; ablation of complex fractionated atrial electrograms (CFAEs), rotors, non-pulmonary foci, or ganglionated plexi; ablation of high dominant frequency sites and fibrosis sites identified by voltage and/or cardiac magnetic resonance imaging (MRI) mapping; and even alcohol injection in the vein of Marshall may be necessary.^2^ As many patients require “multiple” ablations—and for better definition and standardization of clinical terms in daily clinical practice—the term “multiple” ablations should have a universal definition. Another debatable question is whether the atrial tachyarrhythmias that occur post-ablation are due entirely to the proarrhythmic effects of CA or are they part of a continuum of the arrhythmia spectrum from the underlying atriopathy (atrial cardiomyopathy). These questions bear practical patient care–related management consequences.

Herein, we describe a case of a 65-year-old man with multiple comorbidities who presented with AT/left atrial (LA) AFL following “multiple” previous ablations. Attempts at cardioversions were unsuccessful in maintaining the sinus rhythm long term. Given his comorbidities, the choices of AADs were very limited. The patient requested another CA procedure, which was performed using high-density mapping that accurately identified arrhythmia mechanisms, elucidated the disease substrate, and restored the sinus rhythm successfully, resulting in marked clinical improvement upon maintaining the sinus rhythm long term.

## Case presentation

A 65-year-old man with multiple comorbidities, which included coronary artery disease, heart failure (HF), hypertension, diabetes, renal insufficiency, peripheral arterial disease, and a CHA_2_DS_2_-VASc score of 5 points, developed persistent AF in 2008. After initial radiofrequency (RF) CA for PVI, he underwent six more CA procedures over the next 15 years for recurrent AF and other atrial tachyarrhythmias (repeat PVI and CFAE ablation in 2010, PVI with LA roof line in 2011, repeat PVI and posterior wall isolation [PWI] in 2012, mitral valve isthmus line for LA-AFL in 2014, multiple linear lesions for LA-AFL in 2020, and multiple linear lesions for LA-AFL in 2023). The procedures were performed by different operators at various institutions. Although cardiac MRI was never performed, cardiac computerized tomographic (CT) scans were performed for three of the CA procedures to detail the anatomy of the PVs, the left atrium, and other structures. Coupled with multiple echocardiographic imaging studies, including intracardiac echocardiography (ICE), the CT scans demonstrated a severely dilated left atrium and the presence of four normal-sized PVs. Additionally, three-dimensional electroanatomic mapping (EAM) systems (CARTO^®^ [Biosense Webster, Diamond Bar, CA, USA] and EnSite™ NavX™ [Abbott, Chicago, IL, USA]) were used at the discretion of the operators for CA procedures. All CA procedures were performed with RF energy. Standard irrigated catheters, and contact force–sensing catheters were used once they became available in the United States.

**Figure 1:**
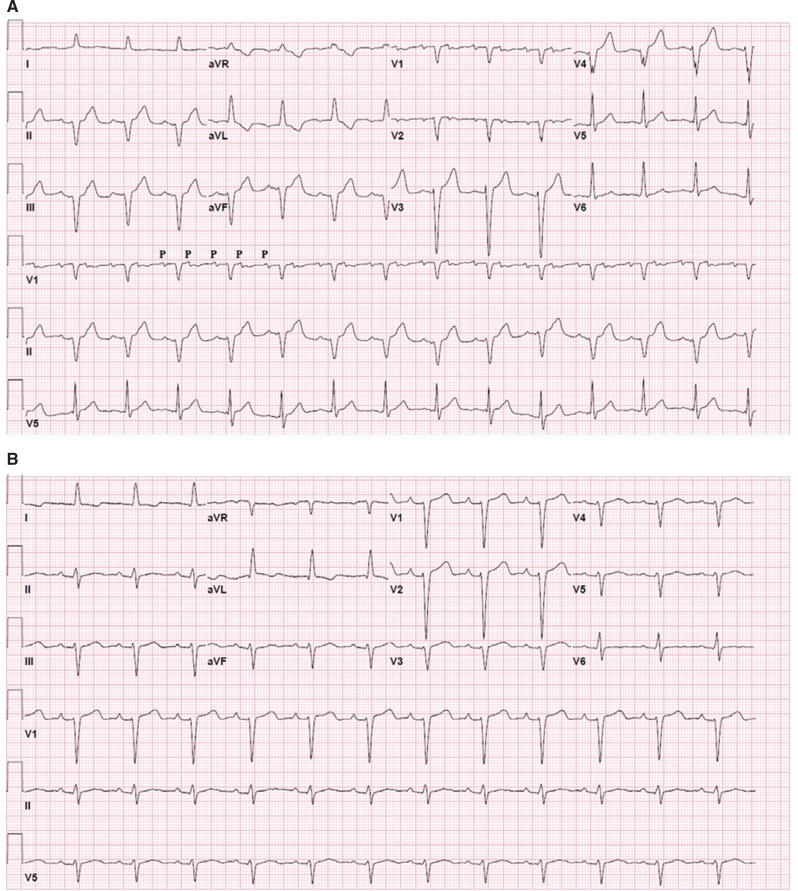
**A:** Pre-ablation electrocardiogram shows atrial tachycardia at a steady cycle length of 390 ms with 2:1 atrioventricular conduction. P-waves are of low amplitude and are labeled. **B:** Post-ablation electrocardiogram shows P-waves in sinus rhythm with 1:1 atrioventricular conduction.

The patient presented with AT/left AFL (**Figure 1**, top panel electrocardiogram [ECG]) despite being treated with amiodarone, which would require interruption due to the elevation of liver enzymes. Given the presence of coronary artery disease and renal insufficiency, other AADs, such as class Ic flecainide and propafenone and class III sotalol and dofetilide, could not be administered. His rhythm was monitored through an implantable loop recorder (ILR). Multiple cardioversions were performed, but long-term sinus rhythm could not be sustained, and the rhythm would revert back to AT/left AFL, resulting in dyspnea and fatigue. His left ventricular ejection fraction (LVEF) had dropped 40%–50%, and the patient continued to have atrial arrhythmias and symptomatic HF.

After multiple shared decision-making discussions that included detailed dialogues about the appropriateness of yet another CA after multiple ablations, the procedural risks, the limitations of pharmacotherapies, and even foregoing all rhythm-control strategies and resorting only to a rate-control approach, the patient requested another CA procedure.

**Figure 2:**
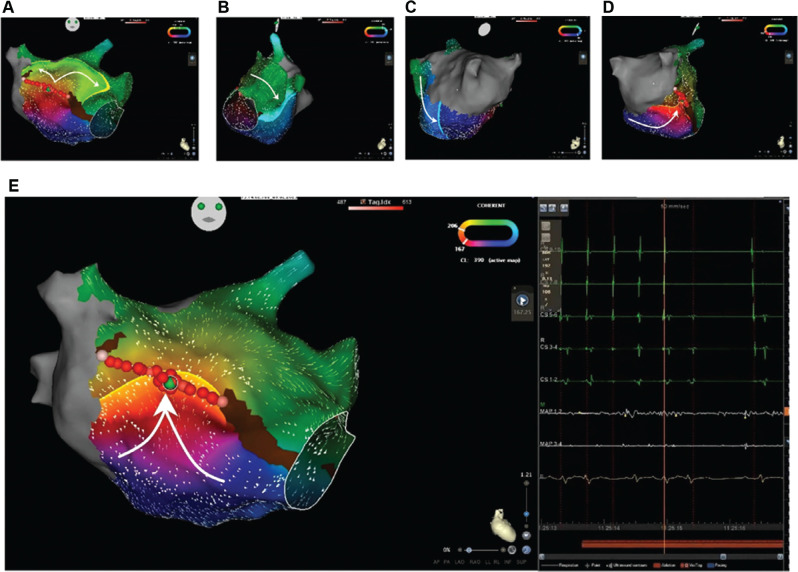
Coherent maps show tachycardia activation (color-coded; red—early to purple—late points) and conduction velocity vectors displaying the electric wave propagation. The top panel includes the entire left atrial chamber divided into **(A)** anterior, **(B)** left lateral, **(C)** posterior, and **(D)** right lateral segments, with white arrows tracking the activation sequence. The bottom left panel **(E)** shows the early-meets-late activation segment between the left atrial anterior wall and the right superior pulmonary vein. The pink, red, and green dots represent the start and end ablation points, ablation lesions, and tachycardia termination point, respectively. The brown line represents the previously ablated anterior mitral isthmus line, which was incomplete. The Coherent tachycardia cycle length loop also confirms the entire 390-ms cycle length confined in the left atrium. The bottom right panel shows intracardiac electrocardiograms recorded in the coronary sinus (CS) and ablation catheter (MAP) during tachycardia and in sinus rhythm upon termination during the application of RF energy, represented by the brown bar at the bottom.

**Figure 3:**
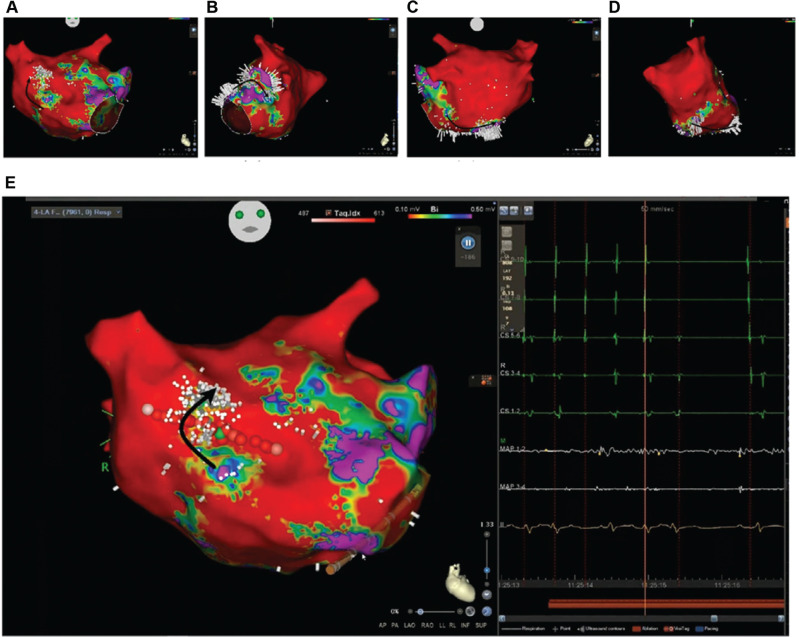
Ripple maps show all components of electrograms (voltage, waveform, and timing) displayed as dynamic bars. Extensive scarring (voltages, ≤0.1 mV; color red) of the posterior wall, roof, and most of the left atrium with low indiscernible and virtually no electrograms and only discrete areas of viable tissue (voltages, 0.25–≥0.5 mV; color yellow/green to purple) within the scarred regions are seen in the anterior wall of the left atrium. The top panel includes the entire left atrial chamber divided into **(A)** anterior, **(B)** left lateral, **(C)** posterior, and **(D)** right lateral segments, with the black arrow tracking the activation sequence. The bottom left panel **(E)** shows the early-meets-late activation segment between the left atrial anterior wall and the right superior pulmonary vein. The pink, red, and green dots represent the start and end ablation points, ablation lesions, and tachycardia termination point, respectively. The brown line represents the previously ablated anterior mitral isthmus line, which was incomplete. The bottom right panel shows intracardiac electrograms recorded in the CS and ablation catheter (MAP) during tachycardia and in sinus rhythm upon termination during the application of radiofrequency energy, represented by the brown bar at the bottom.

The patient’s eighth CA procedure was performed under general anesthesia, with the CARTO^®^ 3 mapping system (version 7.2) (Biosense Webster). Intraoperative ICE imaging demonstrated the presence of an aneurysmal interatrial septum, proximity of the esophagus to the left PV, and pulse-wave Doppler velocities in the PVs at 0.3 m/s and in the LAA at 0.4 m/s. The presenting arrhythmia was AT/left AFL at a steady cycle length of 390 ms with 2:1 atrioventricular conduction (**Figure 1**, top panel, pre-ablation ECG). Intracardiac electrograms (EGMs) showed eccentric atrial activation from the distal to proximal coronary sinus (CS) poles **(Figures 2 and 3)**. Entrainment was deliberately not performed to prevent arrhythmia transformation to other arrhythmias or even termination. An F-curve OCTARAY™ mapping catheter (Biosense Webster) with 3–3–3–3–3-mm interelectrode spacing and TRUEref™ technology for the recording of unipolar EGMs was used to acquire 13,336 points from both atria in <8 min. Along with standard activation and propagation and voltage maps, Coherent and Ripple mapping (Biosense Webster) were performed. Coherent mapping combines activation with color (red to purple) and conduction velocity vectors to display the electric wave propagation. In Ripple mapping, all components of EGMs (voltage, waveform, and timing) are displayed as dynamic bars.

All four PVs were found to be electrically silent. The voltage map of the left atrium showed extensive scarring (voltages ≤0.1 mV, color red, **Figure 3**) of the posterior wall, roof, and most of the left atrium with low indiscernible and virtually no EGMs, and only discrete areas of viable tissue (voltage, 0.25–≥0.5 mV; color yellow/green to purple) within the scarred regions were seen in the anterior wall of the left atrium. Coherent and Ripple maps (**Figures 2 and 3** and **Supplementary Videos 1 and 2**) revealed the presence of a macro–re-entrant AT/left AFL circuit with early-meets-late activation between the LA anterior wall and the right superior PV.

The mapping catheter was then exchanged for a THERMOCOOL SMARTTOUCH™ ablation catheter (Biosense Webster). RF ablation was performed by delivering RF energy at 35 W, maintaining a contact force of 10–15 g at the discontinuous segment of the previous ablation line (**Figures 2 and 3**; pink dots—start and end points, red dots—ablation lesions, green dot—tachycardia termination point). Further lesions were delivered to complete the ablation line. Bidirectional conduction block across the ablation line was seen with marked conduction timing delays on the contralateral aspect of the line with pacing maneuvers. Post-ablation, high-dose intravenous isoproterenol challenge was not performed in view of the presence of the underlying coronary artery disease and stents. On regular clinical follow-ups and ILR rhythm monitoring (10 months since ablation), the patient has remained in sinus rhythm (**Figure 1B**, post-ablation ECG) with marked clinical and LVEF improvement.

### Ethical standards

Ethical standards were observed as per the 1975 Declaration of Helsinki, and the patient was treated as per the prevailing standard of care. Informed consent was obtained from the patient for conducting the ablation procedure. Furthermore, all catheters and software used during the procedure were approved by the U.S. Food and Drug Administration.

## Discussion

Our case report highlights several features of complex arrhythmia management in patients with AF and raises some unresolved questions. For example, how many numerical CA procedures constitute the definition of “multiple” ablations? What is the incidence of recurrence of AF and other atrial arrhythmias after “multiple” ablations? Are there any characteristics of atrial arrhythmias after “multiple” ablations? What is the optimal treatment strategy for recurrent atrial arrhythmias?

Presently, no professional societies have addressed the issue of “multiple” ablations in their guidelines or scientific statements.^1–4^ Nonetheless, from a practical standpoint, three or more CA procedures are considered “multiple” ablations. It is not uncommon for some patients who have undergone PVI ablation for paroxysmal AF to develop recurrence and resort to second, third, or more CA ablation procedures. There are no major randomized controlled trials or multicenter registries detailing multiple AF ablation procedures, but a few single-center studies are worth mentioning. In a study by Lin et al. of 2886 patients who underwent AF ablation from 2000–2012 at the Hospital of the University of Pennsylvania, 6% of patients underwent three procedures, which included 81% undergoing three procedures and 18% undergoing four procedures. Interestingly, at the time of third or greater AF ablation, PV reconnection was the rule (92%), and PV triggers initiating AF could be demonstrated.^5^ In a study from the University Medical Center Utrecht in the Netherlands, among 518 patients who underwent CA from 2005–2011, only 58% attained 5-year freedom of atrial tachyarrhythmias by a single PVI, and 41% of patients underwent multiple procedures.^6^ Among these patients, the incidence of LA-AFL or LA-AT was 5% after PVI and increased to 32% after additional substrate modification. More failures of CA were seen in patients with persistent AF and long-standing persistent AF. Independent predictors for arrhythmia recurrence after the last ablation were non-paroxysmal AF, persistent AF (hazard ratio [HR], 2.19; 95% confidence interval [CI], 1.56–3.06), long-standing persistent AF (HR, 2.94; 95% CI, 1.96–4.39), history of AF (HR, 1.03; 95% CI, 1.01–1.05), female sex (HR, 1.47; 95% CI, 1.04–2.08), body mass index (HR, 1.02; 95% CI, 1.01–1.03), and hypertension (HR, 1.57; 95% CI, 1.13–2.18).^6^ An enlarged left atrium and additional lesions beyond PVI performed during the index procedure have also been identified as predictors of early and late LA-AFL and LA-AT after CA of AF.^7^ The issue of occurrence of LA-AFL and LA-AT has not diminished with the availability of more advanced technologies in performing CA in the recent contemporary years. In the Catheter Ablation for Persistent Atrial Fibrillation: A Multicenter Randomized Trial of Pulmonary Vein Isolation (PVI) vs. PVI with Posterior Left Atrial Wall Isolation (CAPLA) study, which was a randomized trial of PVI alone versus PVI with PWI in patients with persistent AF, there was no difference in freedom from atrial arrhythmia with/without AADs after multiple procedures (58.2% for PVI with PWI vs. 60.1% for PVI alone [HR, 1.10; 95% CI, 0.79–1.55]), freedom from symptomatic AF with/without AADs after multiple procedures (68.2% vs. 72% [HR, 1.20; 95% CI, 0.80–1.78]), or AF burden (0% in either group).^8^ In terms of arrhythmia mechanisms of LA-AFL and LA-AT, a study involving ultra–high-resolution activation mapping with or without entrainment in a total of 132 patients showed anatomic macro–re-entry in 60%, scar-related localized re-entry in 27%, and focal AT in 9% of patients. A total 45% of patients had multiple ATs (20% dual-loop re-entry; 43% sequential AT) with complex and highly variable transitions between AT circuits.^9^

Our case report also brings in the discussion on rhythm-control over rate-control treatment strategies in patients with AF. With lower mortality and stroke or acute coronary syndrome in patients with AF treated with a rhythm-control regimen, the benefits of an early rhythm-control regimen were confirmed in a multicenter randomized trial, Early Treatment of Atrial Fibrillation for Stroke Prevention Trial (EAST-AFNET 4).^10^ In EAST-AFNET 4, the inferiority of AADs as a rhythm-control strategy became obvious as more patients who were started initially on AADs in rhythm control switched to CA at 2 years, as follows: amiodarone from 19.6% to 11.8%, dronedarone from 16.7% to 5.9%, flecainide from 35.9% to 21.0%, propafenone from 7.0% to 3.8%, and other unclassified AADs from 7.6% to 3.2%. Thus, the overall superiority of the rhythm-control regimen observed in EAST-AFNET 4 was due to the inclusion of CA for rhythm control in the trial. As seen with many patients in clinical practice, our patient was also unable to take AADs; amiodarone was discontinued due to the elevation of liver enzymes, and class Ic flecainide and propafenone and class III sotalol and dofetilide were contraindicated due to the presence of coronary artery disease and renal insufficiency, respectively.

As against the lack of newer AADs, the non-pharmacological arena has progressed tremendously over the last two decades.^4^ It is now possible to perform sophisticated and high-density mapping very quickly with newer mapping systems. Even implantable cardiac devices have given newer insights to our understanding of AF and its arrhythmia substrate. Observations such as a decline in the atrial EGM amplitudes over a period of time independent of age and sex in patients with substrate for AF with implanted defibrillators raise a possibility of the development of subclinical and progressive atrial myopathy in such patients.^11^ Multimodality imaging methods, including late gadolinium enhancement on cardiac MRI scans to visualize and assess scars even in thin anatomical structures such as the atria, have enhanced the ablation strategies. The late gadolinium enhancement levels of 3, 4, and 5 standard deviations above the blood pool mean value have been shown to closely associate with progressively lower bipolar voltages on both EnSite™ NavX™ and CARTO^®^ 3 mapping systems.^12^ Findings of significant impairment of active LA function and its correlation with LA scar as assessed by cardiac MRI and voltage mapping by EAM in patients subjected to multiple CAs compared to ablation-naïve patients suggest that CA per se results in increased LA scar burden with consecutively impaired active LA function.^13^ However, in the absence of large studies with serial measurements of atrial scars and function, one cannot completely disregard the role of underlying atriopathy contributing to the atrial scar and deteriorating function.

As regards the mapping and ablation strategy, the role of high-density mapping, such as the CARTO^®^ 3 system’s Coherent and Ripple mapping, and abstaining from the entrainment technique to define a macro–re-entrant circuit, as in our case, merit discussion. In a study by Vicera et al. that involved 26 complex ATs in 20 patients, Coherent mapping with conduction velocity vectors derived from the adjacent mapping sites significantly improved the identification of critical isthmus sites in scar-related ATs better than conventional mapping. With Coherent mapping, ablation terminated the tachycardia (96.2% vs. 69.2%; *P* = .010) and identified significantly more critical isthmuses (mean/chamber, 2.0 ± 1.1 vs. 1.0 ± 0.7; *P* < .001) with narrower widths (19.8 ± 10.5 vs. 43.0 ± 23.9 mm; *P* < .001) than conventional mapping.^14^ Likewise, in a study by Katritsis et al. that involved 28 patients with LA-AFL (including 26 patients with prior AF ablation), Ripple mapping correctly identified critical scar-related isthmus in 12 out of 28 patients. Ripple mapping–guided ablation terminated the tachycardia in all 12 patients (100%) compared to 10 of 16 patients (63%) treated with empirical, anatomic ablation (*P* = .027) and took less ablation time (11.4 ± 5.3 vs. 26.2 ± 17.1 min; *P* = .0004).^15^ Given the shortcomings of entrainment, ie, misdiagnosis, deterioration of arrhythmias, and even termination, we do not routinely utilize entrainment mapping in our ablation strategy. Recently, Zhang et al. showed similar 1-year outcomes of freedom from atrial tachyarrhythmias after CA of complex AT guided only by high-density mapping in 40 patients compared to 27 patients who underwent entrainment mapping. On the contrary, in four patients, there was an undesired termination of AT during entrainment.^16^

Our case report also touches upon another debatable question regarding whether the atrial tachyarrhythmias that occur post-ablation are due entirely to the proarrhythmic effects of CA or are they part of a continuum of the arrhythmia spectrum from the underlying atriopathy (atrial cardiomyopathy). Very extensive scarring beyond PV antral and other conventional ablation areas that we recorded with EAM favors the notion of a continuum of the arrhythmia substrate from the underlying atriopathy.

Finally, the double jeopardy that incurs from AF and all scales of HF is well known. On a broader scale, well-designed randomized controlled trials and non-randomized studies of CA in HF patients have shown beneficial effects of CA as a rhythm-control strategy in patients with HF with improvements in the symptoms and clinical status of HF.^17^ Appropriate patient-selection criteria are vitally important for such beneficial outcomes, which are not seen universally due to inappropriate patient selection. Young patients with paroxysmal AF, fewer comorbidities, and early-stage HF are the ideal candidates for CA. On the contrary, CA is perhaps not ideal for patients who are older and frail, those who have multiple comorbidities like diabetes and renal insufficiency, and those with non-paroxysmal chronic persistent AF that has already progressed to cause marked atriopathy.^17^

## Conclusion

Our case report highlights several features of complex arrhythmia management in patients with AF, many of whom undergo repeated CA in their lifetime. Based on our learning from our case and from the available data in the literature, as there is no guideline-based limit to the number of CA procedures, the decision to pursue another CA needs to be made on an individual basis and using a shared decision-making process. Furthermore, on the basis of our experience from the case, we believe that, when CA is undertaken, the procedure should be performed using high-density mapping to facilitate accurate identification of the arrhythmia mechanisms and successful restoration and long-term maintenance of sinus rhythm.

## Figures and Tables

**Video 1:** Video of the Coherent map show tachycardia activation (color-coded, red-early to purple-late points) and conduction velocity vectors displaying the electric wave propagation in the left atrial chamber with white arrows tracking the activation sequence. The early-meets-late activation segment is between the left atrial anterior wall and the right superior pulmonary vein. The pink, red, and green dots represent start and end ablation points, ablation lesions, and tachycardia termination point, respectively. A brown line represents a previously ablated anterior mitral isthmus line, which was incomplete. The Coherent tachycardia cycle length loop also confirms entire 390=ms cycle length confined in the left atrium.

**Video 2:** Video of the Ripple maps show all components of electrograms (voltage, waveform, and timing) displayed as dynamic bars. Extensive scarring (voltages ≤0.1 mV, color red) of the posterior wall, roof and most of the left atrium with low indiscernible and virtually no electrograms and only discrete areas of viable tissue (voltages 0.25 mV–≥0.5 mV, color yellow/green to purple) within the scarred regions are seen in the anterior wall of the left atrium. The pink, red, and green dots represent start and end ablation points, ablation lesions, and the tachycardia termination point, respectively.
